# Conserved Phosphoprotein Networks Identify Actionable Adhesion/Wnt and Metallothionein Modules in Cholangiocarcinoma

**DOI:** 10.3390/medsci14010063

**Published:** 2026-01-30

**Authors:** Sirinya Sitthirak, Sittiruk Roytrakul, Arporn Wangwiwatsin, Nisana Namwat, Poramate Klanrit, Hasaya Dokduang, Prakasit Sa-ngiamwibool, Attapol Titapun, Apiwat Jareanrat, Vasin Thanasukarn, Natcha Khuntikeo, Teh Bin Tean, Luke Boulter, Yoshinori Murakami, Watcharin Loilome

**Affiliations:** 1 Department of Medical Technology, School of Allied Health Sciences, Walailak University, 222 Thaiburi, Thasala District, Nakhon Si Thammarat 80161, Thailand; sirinya.sit@wu.ac.th; 2 Research Excellence Center for Innovation and Health Products (RECIHP), Walailak University, Nakhon Si Thammarat 80160, Thailand; 3 National Center for Genetic Engineering and Biotechnology (BIOTEC), National Science and Technology Development Agency (NSTDA), Pathum Thani 12120, Thailand; sittiruk@biotec.or.th; 4 Department of Systems Biosciences and Computational Medicine, Faculty of Medicine, Khon Kaen University, Khon Kaen 40002, Thailand; arpowa@kku.ac.th (A.W.); nisana@kku.ac.th (N.N.); porakl@kku.ac.th (P.K.); 5 Cholangiocarcinoma Research Institute, Khon Kaen University, Khon Kaen 40002, Thailand; hasaya.d@msu.ac.th (H.D.); attati@kku.ac.th (A.T.); apiwat.apj@kku.ac.th (A.J.); maxpasin34@gmail.com (V.T.); natckh@kku.ac.th (N.K.); 6 Faculty of Medicine, Mahasarakham University, Mahasarakham 44000, Thailand; 7 Department of Pathology, Faculty of Medicine, Khon Kaen University, Khon Kaen 40002, Thailand; prakasa@kku.ac.th; 8 Department of Surgery, Faculty of Medicine, Khon Kaen University, Khon Kaen 40002, Thailand; 9 National Cancer Centre Singapore, Duke-NUS Medical School, Singapore 169857, Singapore; gmstbt@nus.edu.sg; 10 MRC Human Genetics Unit, Institute of Genetics and Cancer, The University of Edinburgh, Western General Hospital, Crewe Road South, Edinburgh EH4 2XU, UK; luke.boulter@ed.ac.uk; 11 Department of Molecular Biology, Institute for Advanced Medical Sciences, Nippon Medical School, Tokyo 113-8602, Japan; yoshinori-murakami@nms.ac.jp

**Keywords:** cholangiocarcinoma, phosphoproteomics, proteomics, LC-MS/MS, signaling pathways, Wnt/β-catenin signaling, drug-protein interaction network

## Abstract

**Background/Objectives:** Cholangiocarcinoma (CCA) is a very aggressive biliary carcinoma characterised by significant molecular heterogeneity and restricted treatment alternatives. Despite genomic and proteomic investigations revealing recurrent changes, the signalling dynamics influencing tumour behaviour remain inadequately comprehended. **Methods:** We conducted high-resolution Liquid Chromatography–Tandem Mass Spectrometry (LC–MS/MS)-based phosphoproteomics on paired tumour and surrounding tissues from 13 CCA patients in Northeast Thailand, meticulously sampling four geographically unique tumour areas for each patient. Our analysis concentrated on phosphoproteins consistently identified across all regions, delineating strong tumour-specific and cohort-wide phosphorylation signatures. **Results:** Notwithstanding considerable inter-patient variability, two conserved signalling modules were identified: an adhesion/Wnt axis regulated by hyperphosphorylated CTNNB1 protein (β-catenin) and a metal-handling module facilitated by metallothionein-1G (MT1G) protein and metallothionein-2A (MT2A) protein. Pathway enrichment identified focal adhesion, ECM-receptor interaction, cytoskeletal modulation, and mineral absorption as critical activities. **Conclusions:** This study elucidates conserved oncogenic pathways by analysing phosphoproteomic signatures across regional and patient-level variability, emphasising phosphoproteomics as a robust framework for biomarker and therapeutic development in CCA.

## 1. Introduction

Cholangiocarcinoma (CCA) is a severe cancer of the biliary tract, marked by delayed clinical presentation, limited therapeutic alternatives, and an increasing global prevalence [1,2]. The burden is more pronounced in Northeast Thailand, where the condition exhibits one of the highest incidence rates globally, primarily due to chronic infection with the liver fluke *Opisthorchis viverrini*, dietary nitrosamine exposure, and ongoing biliary inflammation. These elements jointly promote carcinogenesis, leading to unfavourable outcomes and highlighting the pressing need for enhanced molecular comprehension to guide therapeutic approaches [3,4,5]. Despite extensive genomic and proteomics studies identifying recurrent alterations and etiological subgroups, such as fluke-associated disease, the application of these findings to achieve consistent clinical benefits has been limited, with inter- and intratumoural heterogeneity posing significant challenges. Comprehensive genomic and proteomic analyses have revealed recurring mutations, causal subgroups, and anomalous protein expression patterns, including those linked to fluke-associated disease. Nonetheless, the application of these findings to achieve reproducible therapeutic advantages has been constrained, mostly due to significant inter- and intratumoural variability [6,7,8,9]. Profiles frequently vary not just among patients but also between different tumour locations, hindering the identification of accurate biomarkers or universal treatment targets. Addressing this intricacy is essential for the advancement of precision medicine in CCA.

Oncogenic signalling in CCA is regulated not only by protein levels but also by post-translational modifications that influence protein function, assembly, and spatial organisation. Phosphorylation serves as a principal regulatory mechanism, controlling essential processes like adhesion, Wnt/β-catenin signalling, cytoskeletal remodelling, DNA damage response, and metal-ion homeostasis [10,11]. Phosphorylation changes can drive malignant phenotypes independently of alterations in total protein levels, making phosphoproteomics indispensable for capturing functional activation states that influence tumour behaviour and therapeutic response [12]. Despite the potential of phosphoproteomic profiling, previous investigations in hepatobiliary cancers have been constrained by limited phosphoproteome depth, frequent reliance on single-region tumour biopsies, low phosphopeptide stoichiometry, enrichment bias during phosphopeptide isolation, stochastic precursor selection in data-dependent acquisition, and modest cohort sizes. Many studies have depended on single-region sampling, resulting in inadequate representations of the tumour phosphoproteome, and cohort sizes have typically been modest, restricting broader applicability across cohorts. Importantly, few analyses have effectively differentiated between tumour-wide phosphorylation programs and site-specific variations, a distinction that is particularly significant in CCA, where considerable heterogeneity and persistent inflammation prevail in the tumour microenvironment [13,14]. Consequently, the field/research area lacks a comprehensive, cohort-level representation of the CCA phosphoproteome that considers both geographic and inter-patient heterogeneity.

This study sought to overcome these limitations by employing high-resolution Liquid Chromatography–Tandem Mass Spectrometry (LC–MS/MS) based phosphoproteomics with phosphopeptide enrichment on paired tumour and adjacent non-malignant tissues from patients in Northeast Thailand. Our objective was to identify tumour-specific phosphoproteins reliably present across many spatially diverse locations inside each tumour and preserved among patients through systematic sampling. We additionally aimed to examine the functional roles of these common phosphoproteins and their possible associations with clinically utilised pharmaceuticals. This approach aimed to create a strong and translationally significant phosphoproteomic framework for CCA, providing new avenues for biomarker identification, treatment prioritisation, and future precision oncology efforts.

## 2. Materials and Methods

### 2.1. Collection of Patients and Samples

Fresh-frozen CCA tissues were procured from patients who received curative surgery at Srinagarind Hospital, Faculty of Medicine, Khon Kaen University. The multi-region sampling technique and patient enrolment criteria were previously delineated in our proteomics investigation on CCA tumour heterogeneity (Proteomic profiling identifies common and region-specific protein signatures underpinning tumour heterogeneity in CCA). Thirteen patients who satisfied the surgical criteria submitted written informed consent, and tumour samples together with neighbouring non-malignant regions were obtained. A total of 65 tumour locations were collected, resulting in 52 high-quality specimens for subsequent proteome and phosphoproteomic analysis. All procedures involving human participants and human tissue samples adhered to pertinent rules and legislation, including the Declaration of Helsinki. The Ethics Committee for Human Research at Khon Kaen University, Thailand, examined and approved the study protocol (Approval No. HE671356; date of approval 5 June 2025).

### 2.2. Protein Extraction, Phosphoprotein Enrichment, and Label-Free Protein Quantification via Mass Spectrometry Sample Preparation for Shotgun Proteomics

Phosphoproteome profiling involved the enrichment of phosphopeptides using a Pierce^®^ Phosphoprotein Enrichment Kit (Thermo Fisher Scientific, Waltham, MA, USA). One hundred micrograms of protein were utilised for phosphoprotein enrichment via the Pierce^®^ Phosphoprotein Enrichment Kit following the manufacturer’s guidelines and subsequently desalted utilising HiTrap^®^ Desalting Columns (Merck KGaA, Darmstadt, Germany). The phosphoproteins underwent reduction with 10 mM dithiothreitol (DTT) and alkylation with 30 mM iodoacetamide (IAA) in 10 mM ammonium bicarbonate, then were subsequently digested with sequencing-grade trypsin (Promega Corporation, Madison, WI, USA) for 16 h at 37 °C. Tryptic peptides were subsequently concentrated with a SpeedVac™ Vacuum Concentrator (Thermo Fisher Scientific, Waltham, MA, USA) and reconstituted in 0.1% formic acid (FA) for mass spectrometry analysis.

### 2.3. Liquid Chromatography–Tandem Mass Spectrometry (LC–MS/MS)

The tryptic peptide samples were prepared for injection into an Ultimate3000 Nano/Capillary LC System (Thermo Scientific, Waltham, MA, USA) linked to a Hybrid quadrupole Q-Tof Impact II mass spectrometer (Bruker Daltonics, Bremen, Germany) which included a nano-electrospray ionisation source. A volume of 1 microlitre from each peptide digest was concentrated utilising a C18 trap column (300 μm × 5 mm, 5 μm particle size, 100 Å pore size from Thermo Scientific, Waltham, MA, USA). The peptides were further separated utilising a 75 μm × 15 cm C18 nano-analytical column composed of 2 μm, 100 Å particles, specifically the Acclaim PepMap RSLC column produced by Thermo Scientific. The column temperature was sustained at 60 °C. The mobile phases A and B consisted of 0.1% formic acid in water and 0.1% formic acid in 80% acetonitrile, respectively. The elution of peptides was conducted with a linear gradient of solvent B, varying from 5% to 55% over a period of 30 min. The flow rate for this procedure was established at 0.30 μL/min. Electrospray ionisation was performed at a voltage of 1.6 kilovolts using the CaptiveSpray source. The drying gas employed was nitrogen, with a flow rate of around 50 L per hour. Product ion spectra were acquired by collision-induced dissociation (CID), with nitrogen as the collision gas. Mass spectrometry (MS) and tandem mass spectrometry (MS/MS) data were acquired in positive ion mode at a frequency of 2 Hz, encompassing the mass-to-charge ratio (*m*/*z*) range of 150–2200. The collision energy was incrementally raised to 10 electron volts (eV) in relation to the mass-to-charge (*m*/*z*) ratio. The nanoLC-MS/MS technology was employed to evaluate each sample three times.

### 2.4. Bioinformatics and Data Analysis

The MaxQuant 2.2.0.0 software was utilised to quantify the phosphoproteins in each sample. The Andromeda search engine integrated within MaxQuant was employed to align the acquired tandem mass spectra (MS/MS) with the Uniprot protein database for *Homo sapiens* [15]. This enabled the identification and quantification of proteins in the samples according to the defined peptide sequences. Standard MaxQuant parameters were employed for label-free quantification, permitting a maximum of two missed trypsin cleavages and a mass tolerance of 0.6 Da for the initial search. Trypsin was identified as the enzyme responsible for digestion. The fixed modification was the carbamidomethylation of cysteine, while the variable modifications included the oxidation of methionine and N-terminal acetylation of the protein. In the phosphoproteome analysis, the phosphorylation of serine, threonine, and tyrosine residues (Phospho (STY)) and the deamidation of glutamine and asparagine (Deamidation (NQ), Gln → Pyro-Glu) were utilised as additional fixed modifications. The criterion for protein identification mandated a minimum of seven amino acids per peptide, at least one peptide unique to each protein, and a minimum of two peptides, with at least one being unique, for each identified protein. A false discovery rate (FDR) of 1% was used at the protein level, established through a search employing a decoy reverse sequencing database. The peptide was restricted to a maximum of five alterations. The Uniprot *Homo sapiens* reference proteome database was employed for the database search.

### 2.5. Data Analysis in Phosphoproteomics

**Figure 1:**
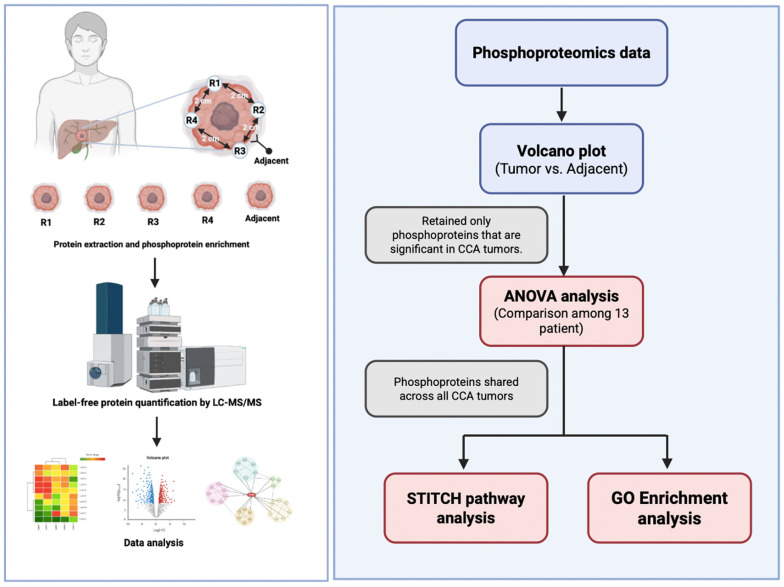
Experimental design and analytical framework of phosphoproteomics in cholangiocarcinoma (CCA). Tumour tissues were obtained from four spatially different regions (R1–R4) and adjacent non-tumorous tissue from each CCA patient. Proteins were isolated and phosphoproteins were enriched before high-resolution Liquid Chromatography–Tandem Mass Spectrometry (LC–MS/MS) based phosphoproteomics analysis. The phosphoproteomic data were analysed for differential expression by comparing tumour and surrounding tissues by volcano plot analysis. Only phosphoproteins that were significantly elevated in CCA tumours were retained for further studies. Inter-patient variability among the 13 persons was evaluated using one-way ANOVA, and phosphoproteins consistently present in all tumours were identified. The conserved tumour phosphoproteins were further analysed for functionality by protein-drug interaction mapping utilising STITCH and biological pathway enrichment employing the GO and KEGG databases.

The PhosphoproteinGroups.txt file generated by MaxQuant was imported into Perseus version 1.6.6.0. Entries classified as backward, potential contaminants, or solely identifiable by site were omitted. Intensities underwent log_2_ transformation and were normalised across samples. Missing values were considered nonexistent; no imputation was employed for defining the common tumour collection. For studies requiring complete matrices (e.g., visualisation), missing values were imputed in Perseus using a left-censored approach as described in the figure legends. Statistical analysis and data visualisation, including volcano plots and one-way ANOVA for the 13 patients, were performed using MetaboAnalyst 6.0 [16]. Functional annotation was conducted via ShinyGO (v0.82) for KEGG/GO/Reactome enrichment analysis [17,18,19], utilising the comprehensive quantified phosphoproteome as a framework and documenting annotations/results that satisfied Benjamini–Hochberg false discovery rate control standards. Chemical–protein interaction networks were established in STITCH (v5.0; Homo sapiens; minimum required score ≥0.7; evidence sourced from publications and databases; disconnected nodes excluded), as depicted in Figure 1.

## 3. Results

### 3.1. Patient Characteristics

This cohort included patients who had resectable surgery at Srinagarind Hospital, as outlined in Table 1. Tumour specimens were obtained from 13 CCA patients, with four tissue slices collected from each individual, yielding a total of 52 samples, together with adjacent non-tumorous tissues. The patients’ ages ranged from 53 to 77 years, with a mean age of 64 years. The group consisted of seven males and six females. Postoperative survival times varied from 54 to 1694 days, with an average of 755 days. The outcomes of haematoxylin and eosin (H&E) staining for each sampled region are displayed in Supplementary Table S1.

### 3.2. Differential Phosphoprotein Expression Highlights Tumour-Specific Signatures

**Figure 2:**
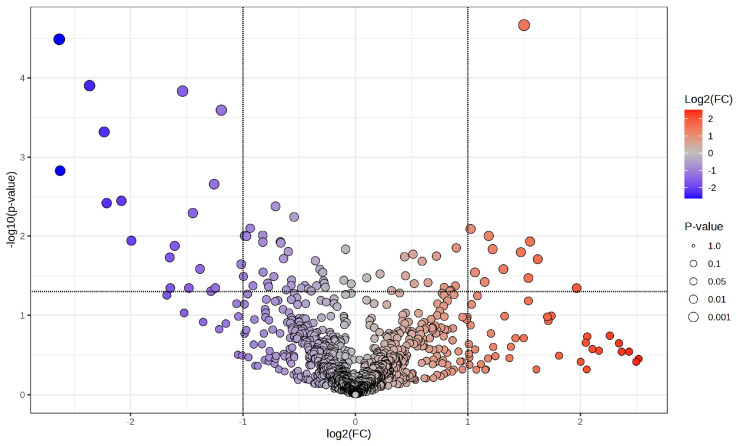
Volcano plot illustrating differential phosphoprotein expression between CCA tumours and adjacent non-tumorous tissues. A volcano graphic illustrating phosphoproteins determined by LC-MS/MS, contrasting cancer tissues with corresponding adjacent non-tumorous tissues. The x-axis illustrates the log_2_ fold change (log_2_FC) in phosphoprotein abundance, while the y-axis indicates the log_10_(*p*-value) derived from statistical analysis. Phosphoproteins that are enriched in tumours (log_2_FC > 1, −log_10_*p* > 1.3) are shown on the right (red gradient), while those that are depleted in tumours (log_2_FC < −1, −log_10_*p* > 1.3) are illustrated on the left (blue gradient). Grey points denote non-significant features. The dimensions of each point correspond to the significance level (*p*-value). This investigation identifies a group of phosphoproteins that are particularly prevalent in cancer tissues, many of which are associated with critical oncogenic pathways, indicating their potential as biomarkers and therapeutic targets in cholangiocarcinoma.

A volcano plot (Figure 2) illustrates the distribution of phosphoproteins measured between cancer and corresponding surrounding tissues, emphasising those specifically identified in tumour samples. The x-axis represents the log_2_ fold change (log_2_FC) in abundance between tumour and neighbouring tissue, while the y-axis illustrates the –log_10_(*p*-value) derived from statistical comparison. Phosphoproteins that are abundant in tumours (log_2_FC > 1, –log_10_(*p*-value) > 1.3; right quadrant, red gradient) and those that are deficient in tumours (log_2_FC < –1, –log_10_(*p*-value) > 1.3; left quadrant, blue gradient) are distinctly differentiated from non-significant characteristics (grey). The size of the points indicates statistical significance, with larger points representing lower *p*-values. This investigation identifies a specific subset of phosphoproteins found only in cancer tissues, many of which are involved in critical oncogenic processes, indicating their potential as biomarkers or therapeutic targets in CCA.

### 3.3. Inter-Patient Heterogeneity of Tumour-Specific Phosphoproteins in CCA

**Figure 3:**
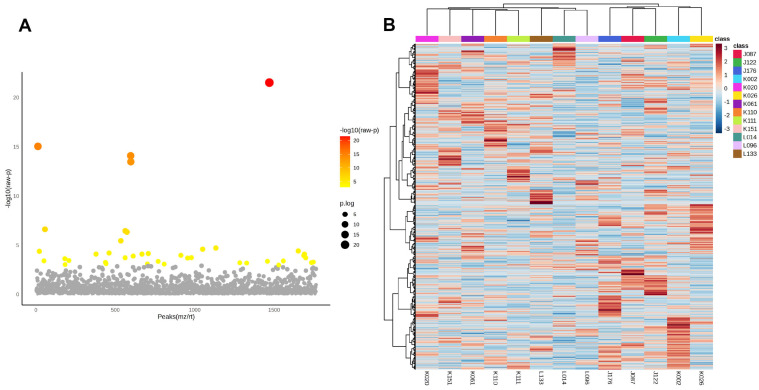
Inter-patient variability of tumour-specific phosphoproteins in cholangiocarcinoma. (**A**) One-way ANOVA analysis of phosphoproteins in 13 CCA patients. The x-axis denotes phosphoprotein peaks (*m*/*z*/*rt*) and the y-axis illustrates the −log_10_(raw *p*-value). Each dot represents a phosphoprotein, with statistical significance denoted by colour (a gradient from yellow to red indicates increasing significance; grey indicates non-significance). Significant phosphoproteins exhibit considerable variation in abundance among patients, highlighting strong inter-patient heterogeneity within the tumour phosphoproteome. (**B**) Hierarchical clustering heatmap of significantly changing phosphoproteins among patients. Columns denote specific patients, whereas rows signify phosphoproteins. The colour gradient indicates relative abundance (red = greater, blue = lesser). Distinct clustering patterns underscore unique phosphorylation signatures among various patients, supporting the presence of patient-specific subgroups within the sample. The data indicate that, despite common tumour-specific phosphoproteins, CCA displays significant biological variety in phosphorylation, potentially leading to therapeutic variability and treatment resistance.

After identifying tumour-specific phosphoproteins, a one-way ANOVA was conducted to evaluate variations in phosphoprotein abundance among the 13 CCA patients. Panel A (Figure 3A) of the volcano plot illustrates the distribution of statistically significant features, where colour intensity indicates –log_10_(raw *p*-values) and point size denotes the degree of significance. Numerous phosphoproteins showed significant diversity among patients, highlighting substantial inter-patient variation within the tumour-specific phosphoproteome.

Panel B (Figure 3B) presents a heatmap that illustrates the hierarchical clustering of significantly variable phosphoproteins, highlighting unique expression patterns particular to each patient. These trends emphasise the existence of subgroups within the cohort, highlighting the biological variation in phosphorylation-mediated signalling among CCA patients.

In light of this heterogeneity, we subsequently aimed to identify phosphoproteins consistently observed in all individuals. The common tumour phosphoproteins, detailed in Supplementary Table S1, exemplify resilient and recurrent molecular characteristics that surpass individual diversity. They may represent pivotal candidates for translational research, encompassing the formulation of broad-spectrum therapy methods and biomarker identification in CCA.

### 3.4. Protein–Protein Interaction (PPI) Network of Common Tumour Phosphoproteins Integrated with Clinically Used CCA Drugs

**Figure 4:**
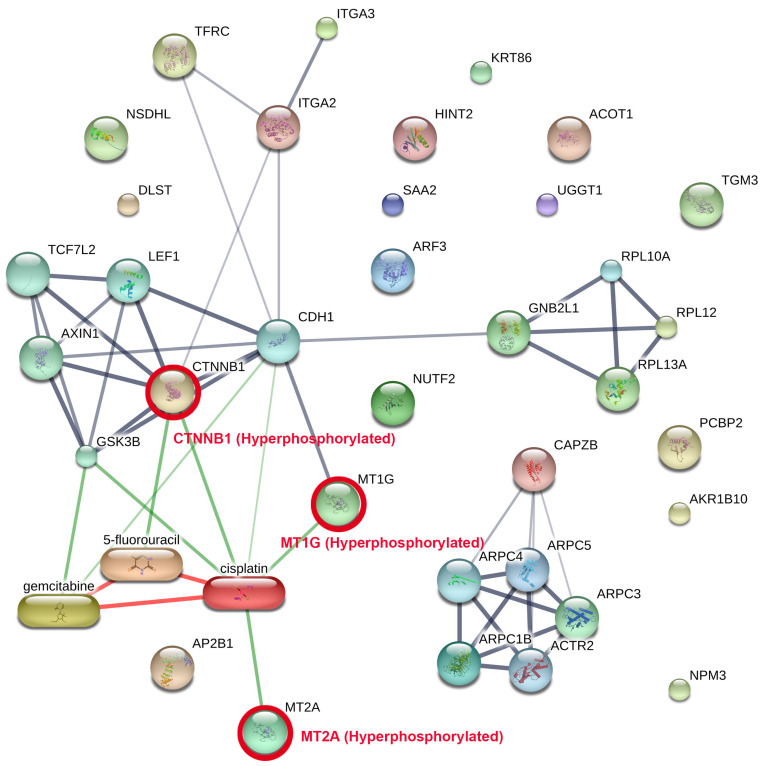
Network of protein–protein interactions (PPI) among common tumour phosphoproteins combined with clinically utilised CCA pharmaceuticals. The network was developed with STITCH, incorporating phosphoproteins reliably recognised across all CCA tumours alongside established drug–protein interactions. Two principal functional modules were identified: (i) an adhesion/Wnt signalling cluster centred on hyperphosphorylated CTNNB1 in conjunction with cadherin and Axin1 complexes, and (ii) a metal-handling module characterised by hyperphosphorylated MT1G and MT2A. These modules were associated with chemotherapeutic drugs employed in the treatment of CCA including cisplatin, gemcitabine, and 5-fluorouracil, with cisplatin functioning as a principal hub connecting the axes. The thickness of the lines indicates the level of confidence in interactions, while red circles emphasise significant hyperphosphorylated proteins. This network demonstrates how phosphorylation-mediated alterations in adhesion/Wnt and metallothionein biology may directly affect drug response, highlighting potential translational targets for combination treatments.

PPI network analysis was performed via STITCH on the phosphoproteins and the degree of phosphorylation consistently found among all patients (Figure 4, Supplementary Table S1), incorporating known interactions with clinically employed chemotherapeutic drugs for CCA (cisplatin, gemcitabine, and 5-fluorouracil). The network identifies two primary functional modules: (i) an adhesion/Wnt signalling cluster focused on hyperphosphorylated CTNNB1 protein (β-catenin), which is hyperphosphorylated in cancer tissue. (The CTNNB1 gene encodes beta-catenin, a multifunctional protein central to the Wnt signalling pathway and cell adhesion processes.) Module (ii) is a metal-binding module facilitated by metallothionein-1G (MT1G) protein and metallothionein-2A (MT2A) protein, which are similarly hyperphosphorylated. (The MT1G gene encodes metallothionein 1G, a cysteine-rich protein that binds heavy metals like zinc, copper, and cadmium to regulate metal homeostasis and protect cells from oxidative stress. The MT2A gene encodes metallothionein 2A, a low-molecular-weight, cysteine-rich protein that binds heavy metals such as zinc, copper, and cadmium to regulate cellular metal homeostasis and act as an antioxidant.) Drug–protein mapping indicates that cisplatin serves as a pivotal drug-associated hub interconnecting the CTNNB1 and metallothionein modules, including supplementary interactions with gemcitabine and 5-fluorouracil. The relationship topology indicates that these hyperphosphorylated nodes may affect medication responsiveness by altering pathways related to cell adhesion, Wnt/β-catenin signalling, and metal-ion homeostasis.

The findings highlight the translational potential of targeting hyperphosphorylated CTNNB1, MT1G, and MT2A proteins, either directly or through pathway modulation in combination with existing chemotherapeutic regimens, thereby motivating further investigation of phosphorylation-dependent mechanisms underlying drug sensitivity and resistance in CCA.

### 3.5. Functional Enrichment Analysis of Shared Tumour Phosphoproteins

**Figure 5:**
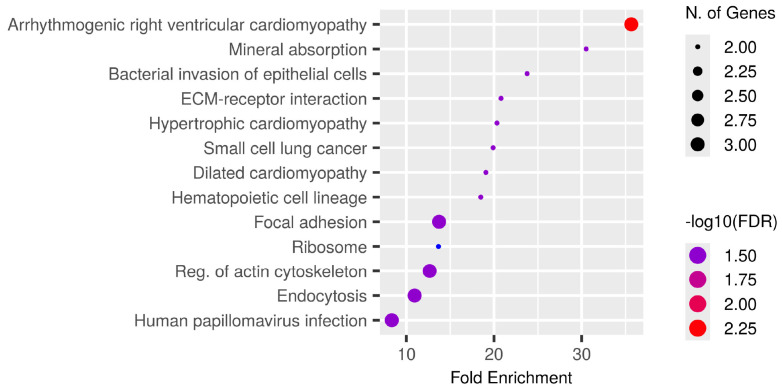
Functional enrichment analysis of phosphoproteins consistently identified among all CCA patients. KEGG pathway enrichment study was conducted on common tumour phosphoproteins. The bubble figure illustrates routes with substantial enrichment, sorted by fold enrichment on the x-axis. The size of the bubble indicates the quantity of related genes and the intensity of the colour represents the significance level (−log_10_FDR). Prominent enriched pathways encompass arrhythmogenic right ventricular cardiomyopathy, mineral absorption, bacterial invasion of epithelial cells, ECM-receptor interaction, focal adhesion, ribosome regulation, actin cytoskeleton modulation, and endocytosis, many of which are integral to cancer-related signalling and tumour microenvironment interactions. The identification of pathways associated with cardiomyopathy underscores the shared cytoskeletal and adhesion mechanisms pertinent to both cardiac and cancer biology, whereas mineral absorption and metallothionein-related processes underscore the significance of metal-ion management in CCA progression. Collectively, these findings indicate that phosphorylation-mediated regulation of adhesion, cytoskeletal remodelling, and ion homeostasis underlies fundamental biological processes in CCA, with implications for biomarker identification and therapeutic intervention.

Phosphoproteins common to all patients (Supplementary Table S1) were subjected to KEGG pathway enrichment analysis, which identifies biological pathways that are statistically overrepresented in a given protein list relative to a background proteome, to investigate their biological and functional context. The bubble plot (Figure 5) illustrates significantly enriched pathways ranked by fold enrichment, where bubble size indicates the number of genes and colour intensity reflects −log_10_(FDR).

The most enriched pathways encompassed arrhythmogenic right ventricular cardiomyopathy, mineral absorption, and bacterial invasion of epithelial cells in addition to significant cancer-related activities including ECM-receptor interaction, focal adhesion, small cell lung cancer, and endocytosis. Multiple signalling pathways crucial to CCA biology, specifically cytoskeletal regulation, ribosomal function, and actin remodelling, were also included.

The findings indicate that the common phosphoproteins are functionally associated with cell adhesion, extracellular matrix interactions, and ion homeostasis, all of which may influence cancer growth and therapeutic response. The enhancement of both cancer-specific and systemic pathways highlights the translational significance of these phosphoproteins for biomarker development and therapeutic targeting in CCA.

### 3.6. Survival Analysis of CTNNB1 and Metallothionein Modules Provides Clinical Context

**Figure 6:**
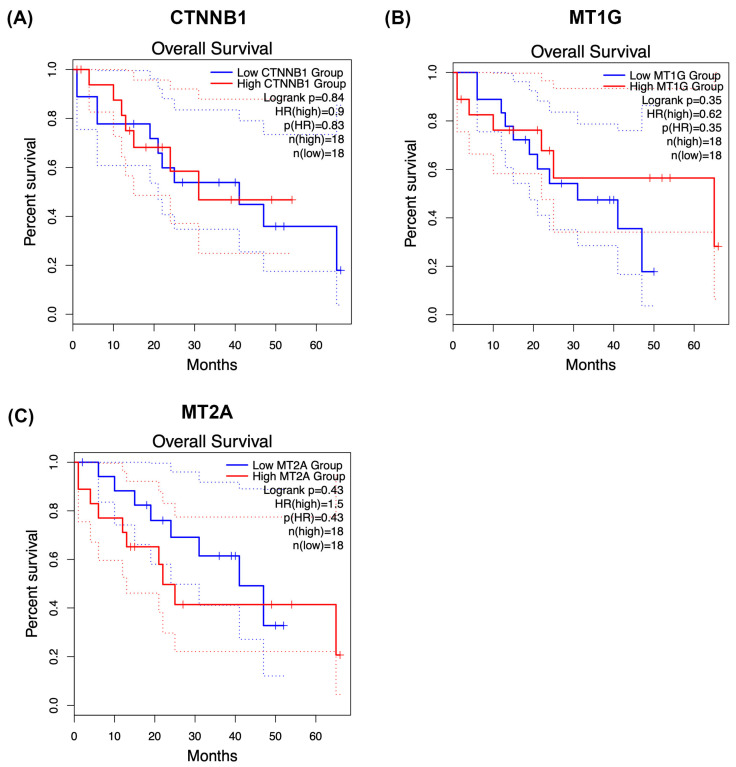
Kaplan–Meier survival analysis of CTNNB1 and metallothionein modules in cholangiocarcinoma. Overall survival was explored in relation to CTNNB1 (**A**), MT1G (**B**), and MT2A (**C**) using Kaplan–Meier analysis. Patients were stratified into high- and low-groups according to median expression or phosphorylation-derived scores, depending on the dataset analysed. Within the phosphoproteomic cohort, each protein exhibited trend-level separation between survival curves. However, none of the comparisons reached statistical significance by log-rank testing, consistent with limited statistical power and biological heterogeneity. Additional exploratory analyses were performed in publicly available expression-based cohorts where feasible to provide clinical context. These results should be regarded as hypothesis-generating, as phosphorylation states may not directly reflect transcript abundance and treatment–response associations could not be robustly assessed. Larger, independent cohorts integrating phosphoproteomics with clinical annotation will be required to determine the prognostic and predictive relevance of these conserved modules in cholangiocarcinoma.

To provide clinical context for the conserved phosphoproteomic modules identified in this study, we explored overall survival associations for CTNNB1, MT1G, and MT2A using Kaplan–Meier analysis (Figure 6A–C). Patients were stratified into high- and low-groups according to median expression or phosphorylation-derived scores, depending on the dataset analysed.

Within our phosphoproteomic cohort, CTNNB1 (Figure 6A), MT1G (Figure 6B), and MT2A (Figure 6C) each showed trend-level separation between survival curves, with higher module activity generally associated with poorer postoperative outcomes. However, none of these comparisons reached statistical significance by log-rank testing, consistent with limited statistical power and pronounced biological heterogeneity in cholangiocarcinoma.

To further contextualise these findings, we additionally examined publicly available expression-based cohorts where feasible (e.g., transcriptomic datasets accessible through KMPlotter or similar resources). These exploratory analyses likewise suggested modest, cohort-dependent trends in survival stratification, but without consistent statistical significance across datasets.

Importantly, these analyses should be regarded as hypothesis-generating. Phosphorylation states are not necessarily mirrored by transcript abundance, and direct treatment–response associations could not be robustly assessed owing to the absence of uniformly annotated therapy-response data in available phosphoproteomic cohorts. Larger, independent studies integrating phosphoproteomics with clinical outcomes and therapeutic response information will therefore be required to determine the prognostic and predictive value of these conserved phosphorylation-dependent modules in CCA.

## 4. Discussion

This study provides a comprehensive phosphoproteomic characterisation of CCA to date, tackling one of the most significant problems in the field: spatial and inter-patient heterogeneity [7,8,20,21]. Through the implementation of a multi-region, cohort-wide approach, we illustrate that phosphorylation-based signalling landscapes can be synthesised into consistent tumour-wide programs, thereby addressing the constraints of previous studies that depended on single-region sampling or limited patient cohorts. Our methodology transforms phosphoproteomics from a mere descriptive tool into a definitive analytical platform, proficient in detecting conserved molecular characteristics that are directly applicable for biomarker development and therapeutic intervention.

The tumour-specific phosphoproteins we found outline key oncogenic pathways in CCA, encompassing adhesion and Wnt/β-catenin signalling, cytoskeletal remodelling, DNA damage response, and metal-ion homeostasis. The phosphorylation events occurred regardless of alterations in total protein expression, highlighting the essential role of phosphoproteomics in identifying functional activation states that influence tumour behaviour [11]. This discovery corroborates previous research indicating that phosphorylation, rather than mere protein quantity, is the principal factor influencing signalling flow and therapeutic response [11,22]. Significantly, our multi-region method demonstrated that whereas each tumour displayed distinct phosphorylation patterns, a core collection of phosphoproteins was consistently maintained across all locations and patients [23]. These compounds serve as durable catalysts of malignant signalling and establish a strong basis for translational research in CCA.

The inter-patient study revealed significant variability, aligning with other genomic studies of CCA that identified various mutational and etiological subgroups [8]. Our research expands this concept to the phosphoproteomic level, demonstrating that variability is not arbitrary but structured into coherent patterns of pathway regulation. We extracted a shared phosphoprotein network from this variety that represents the essential biology of CCA among people. This discovery is particularly pertinent in Isan, where liver fluke infection and chronic inflammation establish a distinctive carcinogenic milieu that influences the tumour microenvironment [1,24,25]. The finding of conserved phosphorylation events in a heterogeneous environment suggests that these proteins may contribute to tumour maintenance and therefore constitute high-priority treatment targets [11,26]. Although the RTK–RAS/MAPK, PI3K–AKT–mTOR, TP53, TGF-β, and Hippo signalling pathways are recognised oncogenic drivers in CCA [1,6] our research aimed not to replace these frameworks but to elucidate phosphorylation programs that are spatially conserved across various tumour regions and patients. Our analytical strategy purposely prioritised robustness and cross-regional consistency by necessitating the recurrent detection of phosphoproteins across geographically diverse locations inside each tumour and throughout the cohort, rather than focussing on pathway-specific sensitivity.

The relatively low detection rate of canonical phosphosignatures, including ERK1/2, AKT, CDK substrates, PRAS40, or 4EBP1, likely results from several non-exclusive factors: significant spatial heterogeneity of kinase activation within CCA lesions; inter-patient variability in pathway utilisation; the transient and low-stoichiometry characteristics of numerous regulatory phosphorylation events; technical biases related to phosphopeptide enrichment and mass spectrometric sampling; and the conservative filtering strategy employed in this study. Consequently, our findings should not be construed as proof of the absence of MAPK- or PI3K-driven signalling in CCA. Instead, they indicate that these pathways did not manifest as universally conserved phosphoproteomic characteristics across the cohort under the rigorous recurrence criteria applied in this study.

In this context, the persistent hyperphosphorylation of CTNNB1 and metallothioneins among various areas and patients should be seen not as evidence of new initiating oncogenic drivers, but as indicative of adaptive signalling modules that endure despite diverse upstream genetic modifications. Metallothioneins have been extensively associated with redox control and metal-ion buffering, perhaps affecting chemotherapy responsiveness rather than cancer initiation. Their preserved phosphorylation across spatially varied tumour sectors underscores their potential significance as functional biomarkers or modulators of treatment sensitivity, particularly in inflammation-prone CCA originating in liver-fluke-endemic areas. Phospho-AKT and various canonical kinase substrates were occasionally observed in specific tumour regions or patients within our dataset. However, they did not meet the rigorous standards for cross-regional consistency and cohort-level reproducibility established in this study, and thus were not prioritised for subsequent network modelling. This discovery aligns with previous extensive phosphoproteomic investigations in CCA, which have documented significant cohort- and platform-dependent variability in pathway activation patterns [20,21,27].

Network-based study revealed that these ubiquitous phosphoproteins cluster into two principal functional modules: an adhesion/Wnt axis centred on hyperphosphorylated CTNNB1 and a metal-handling module centred on hyperphosphorylated MT1G and MT2A. These modules should not be regarded as major oncogenic initiators akin to recurrent genomic drivers like KRAS or TP53, but rather as phosphorylation-regulated signalling programs that are consistently preserved across tumour areas and patients. These conserved modules are, nonetheless, translationally relevant, as they interface with pathways targeted by clinically employed chemotherapeutic drugs [28,29,30]. Cisplatin served as a drug-related connection between both modules, whereas gemcitabine and 5-fluorouracil acted as additional nodes. The results indicate that phosphorylation-dependent regulation of β-catenin/cadherin complexes and metallothionein biology may influence drug sensitivity and resistance [31,32]. Metallothioneins are acknowledged for their functions in redox regulation and metal-ion buffering, potentially serving as stress-adaptive elements that indirectly influence cisplatin efficacy, while Wnt/β-catenin signalling has been linked to chemoresistance in several tumour situations [30,33,34]. These data collectively endorse the advancement of phosphorylation-based biomarkers and strategic combinations while aligning with accepted genetic and inflammatory concepts of CCA aetiology.

The functional enrichment analysis of the shared phosphoproteins emphasised the significance of adhesion, cytoskeletal, and metal-ion regulatory pathways, underscoring activities including focal adhesion, ECM-receptor interaction, endocytosis, and actin remodelling. These pathways are directly associated with invasive and metastatic behaviour, as well as cellular adaptability to stress [35,36,37]. The enrichment of cardiomyopathy-related pathways indicates a significant overlap in adhesion and cytoskeletal signalling between cardiac and cancer biology [38,39]. This discovery suggests that medications initially designed for cardiovascular illness could potentially be explored for repurposing to address similar signalling vulnerabilities in CCA, a concept that merits further investigation.

Collectively, our results establish a phosphorylation-driven disease framework for CCA that is both biologically consistent and applicable to translation. These findings generate hypotheses that necessitate validation in different cohorts and functional perturbation models before causal relationships can be deduced. By establishing discovery through multi-region and multi-patient sampling, we overcame the constraints of previous phosphoproteomics research and unveiled a conserved network of phosphorylation events that delineate the essential signalling architecture of this malignancy [7,40]. These findings have direct translational significance, offering potential biomarkers, drug-related hubs, and treatment approaches based on the dynamic modulation of phosphorylation.

This study illustrates that phosphoproteomics is not only supplementary to genomic and proteomic analysis but is crucial to identifying druggable signalling vulnerabilities in one of the most fatal and treatment-resistant tumours. The proposed methodological framework incorporating spatial resolution, inter-patient consistency, and drug-network mapping provides a template for forthcoming research in CCA and other diverse cancers. By outlining a common phosphoprotein network that surpasses geographical and inter-patient variability, we establish a basis for biomarker identification and therapy development, propelling the field towards precision oncology in CCA. These findings offer a clearer perspective on the phosphorylation landscape in CCA and underscore conserved molecular events that may function as actionable indicators. By tethering hyperphosphorylated CTNNB1 and metallothionein modules to clinically pertinent pharmacological hubs, our research provides a foundation for incorporating phosphoproteomic signatures into patient classification methodologies and biomarker-driven combination treatments. This paradigm facilitates precision oncology strategies in CCA and establishes the groundwork for developing clinical trials that evaluate phosphorylation-based classifiers as indicators of treatment response and resistance. We acknowledge that the relatively modest cohort size and the absence of orthogonal phospho-specific validation constrain the generalisability of our findings and underscore the need for larger, prospective multi-centre studies. No phospho-specific immunoblotting or immunohistochemistry was performed in the present study, which represents an important limitation.

## 5. Conclusions

CCA demonstrates significant geographical and inter-patient heterogeneity, which has hindered the translation of genomic and bulk proteomic findings into dependable biomarkers and widely applicable treatments. This multi-region LC–MS/MS phosphoproteomic investigation of paired tumour and adjacent tissues from 13 CCA patients reveals that phosphorylation-based signalling can be distilled into conserved, tumour-wide programs that endure across geographically distinct tumour regions and among individuals. By selecting phosphoproteins consistently observed across all tumours, we developed a resilient core phosphosignature that reflects functional pathway activity rather than static protein levels.

Notwithstanding significant variability in phosphorylation patterns among patients, network and enrichment analysis identified two conserved and actionable modules: an adhesion/Wnt signalling axis characterised by hyperphosphorylated CTNNB1 and a metal-handling module focused on the hyperphosphorylated metallothioneins MT1G and MT2A. These modules correspond to biologically coherent pathways, including focal adhesion, ECM-receptor interaction, cytoskeletal remodelling, endocytosis, and mineral absorption, emphasising that phosphorylation-dependent regulation of tumour-matrix interactions and metal-ion homeostasis constitutes a common signalling framework in CCA. Significantly, drug–protein interaction mapping identified clinically used chemotherapeutics, especially cisplatin, as central nodes linking these conserved modules, reinforcing the notion that phosphorylation states within β-catenin/adhesion circuitry and metallothionein biology may affect treatment sensitivity and resistance.

Our findings collectively establish a cohort-level, spatially resolved phosphoproteomic framework for CCA, uncovering conserved phosphoprotein networks that can be utilised for biomarker development, patient stratification, and rational combination strategies that integrate pathway modulation with standard chemotherapy. This study highlights phosphoproteomics as a crucial component of precision oncology in CCA, facilitating the identification of functionally druggable vulnerabilities that persist despite significant intratumoural and inter-patient heterogeneity. Significantly, these conserved modules are not suggested as primary oncogenic drivers, but rather as recurring phosphorylation-dependent programs that may influence tumour behaviour and therapeutic response.

## Figures and Tables

**Table 1:** Patient characteristic.

Patient ID	Gender	Age	Survival (Days)
J087	F	65	1694
J122	M	66	663
J176	M	57	322
K002	F	54	369
K020	M	72	1448
K026	F	65	805
K061	M	77	591
K110	F	58	120
K111	M	73	508
K151	M	53	1157
L014	F	68	1121
L096	F	64	969
L133	M	62	54

## Data Availability

The original contributions presented in this study are included in the article. Further inquiries can be directed to the corresponding authors. The raw proteomics data generated and analysed during this study have been deposited in the jPOST (Japan Proteome Standard Repository) under the accession number JPST004071 (https://repository.jpostdb.org/preview/16989698986903555a9ab2f, access key 8191) and PXD068291.

## Supplementary Materials

The following supporting information can be downloaded at: https://www.mdpi.com/article/10.3390/medsci14010063/s1, Table S1: The outcomes of haematoxylin and eosin (H&E) staining for each sampled region.

## Abbreviations

The following abbreviations are used in this manuscript:

CCA	Cholangiocarcinoma
LC–MS/MS	Liquid chromatography–tandem mass spectrometry
ECM	Extracellular matrix
Wnt	Wingless/Integrated signalling
CTNNB1	Catenin beta-1
MT	Metallothionein
MT1G	Metallothionein 1G
MT2A	Metallothionein 2A
PPI	Protein–protein interaction
KEGG	Kyoto Encyclopedia of Genes and Genomes
GO	Gene Ontology
FDR	False discovery rate
CID	Collision-induced dissociation
DTT	Dithiothreitol
IAA	Iodoacetamide
STY	Serine/Threonine/Tyrosine
ANOVA	Analysis of variance
H&E	Hematoxylin and eosin
CVD	Cardiovascular disease
SDP	Single-donor platelet
ROC	Receiver operating characteristic
